# The effects of puberty on white matter development in boys

**DOI:** 10.1016/j.dcn.2014.10.002

**Published:** 2014-10-22

**Authors:** Lara Menzies, Anne-Lise Goddings, Kirstie J. Whitaker, Sarah-Jayne Blakemore, Russell M. Viner

**Affiliations:** a University College London Institute of Cognitive Neuroscience, Alexandra House, 17 Queen Square, London WC1N 3AR, UK; b General Adolescent and Paediatric Unit, University College London Institute of Child Health, 30 Guilford Street, London WC1N 1EH, UK; c Brain Mapping Unit, Department of Psychiatry, Sir William Hardy Building, Downing Street, Cambridge Biomedical Campus, Cambridge CB2 3ED, UK

**Keywords:** Adolescence, Brain development, Puberty, Structural magnetic resonance imaging, White matter, Diffusion tensor imaging, Testosterone

## Abstract

Neuroimaging studies demonstrate considerable changes in white matter volume and microstructure during adolescence. Most studies have focused on age-related effects, whilst puberty-related changes are not well understood. Using diffusion tensor imaging and tract-based spatial statistics, we investigated the effects of pubertal status on white matter mean diffusivity (MD) and fractional anisotropy (FA) in 61 males aged 12.7–16.0 years. Participants were grouped into early-mid puberty (≤Tanner Stage 3 in pubic hair and gonadal development; *n* = 22) and late-post puberty (≥Tanner Stage 4 in pubic hair or gonadal development; *n* = 39). Salivary levels of pubertal hormones (testosterone, DHEA and oestradiol) were also measured. Pubertal stage was significantly related to MD in diverse white matter regions. No relationship was observed between pubertal status and FA. Regression modelling of MD in the significant regions demonstrated that an interaction model incorporating puberty, age and puberty × age best explained our findings. In addition, testosterone was correlated with MD in these pubertally significant regions. No relationship was observed between oestradiol or DHEA and MD. In conclusion, pubertal status was significantly related to MD, but not FA, and this relationship cannot be explained by changes in chronological age alone.

## Highlights

• White matter microstructural differences occurred between early and late puberty.
• White matter regions showed reduced mean diffusivity from early to late puberty.
• Regression models showed that pubertal effects could not simply be ascribed to age.
• Mean diffusivity decreases were associated with increasing salivary testosterone levels.

## 1 Introduction

Cross-sectional and longitudinal structural magnetic resonance imaging (MRI) studies have demonstrated that the human brain undergoes significant development during adolescence (Gogtay et al., 2004, Sowell et al., 2004, Lenroot et al., 2007, Shaw et al., 2008, Giorgio et al., 2010, Raznahan et al., 2011, Ball et al., 2012). These studies have shown that, generally, grey matter decreases in volume during adolescence, with regional variations in precise timing, while white matter volume increases across the brain. The changes occurring at a cellular level that lead to these volume increases are unclear, since neuroimaging lacks the resolution to study this directly. White matter is composed primarily of axons, many of which are myelinated, and associated vasculature and glia. The increases in white matter in adolescence have been proposed to reflect increased axonal calibre within fibre bundles (Paus, 2010) and/or myelination, which in humans continues well into the second and even the third decade of life (Miller et al., 2012).

Diffusion tensor imaging (DTI) provides in vivo quantitative information about white matter microstructure rather than just assessing volumetric changes in white matter (Basser and Pierpaoli, 1996, Le Bihan et al., 2001). Two commonly considered DTI measures are mean diffusivity (MD) and fractional anisotropy (FA) (Basser et al., 1994). MD is a measure of the overall magnitude of water diffusion in any direction, and is sensitive to the number of cells and their processes in a region. In a tight bundle of axons, in which diffusion is restricted due to the large myelin lipids, water diffusion is restricted and MD is low. If the number of cells or cell components (for example myelin, axons or glia) in the region increases, then diffusion will be further restricted and MD will decrease. FA provides information regarding the directionality of diffusion, and represents the extent to which diffusion occurs preferentially in one direction; this measure increases as the extent of axonal myelination increases, or as axons become more coherently organised in a uniform direction (Beaulieu, 2002).

Studies exploring age effects on diffusion indices have consistently identified increases in FA and decreases with MD during adolescence (Klingberg et al., 1999, Morriss et al., 1999, Mukherjee et al., 2001, Schmithorst et al., 2002, Schneider et al., 2004, Barnea-Goraly et al., 2005, Ben Bashat et al., 2005, Snook et al., 2005, Ashtari et al., 2007, Bonekamp et al., 2007, Eluvathingal et al., 2007, Schneiderman et al., 2007, Giorgio et al., 2008, Lebel et al., 2008, Qiu et al., 2008, Tamnes et al., 2010, Bava et al., 2010, Lebel and Beaulieu, 2011, Simmonds et al., 2013). FA increases are generally driven more by reductions in radial diffusion (RD) (in the perpendicular plane to predominant diffusion direction) than by changes in axial diffusion (AD) (in the plane parallel to predominant diffusion direction) (Giorgio et al., 2008, Lebel et al., 2008), although some studies have reported a decrease in both modalities (Eluvathingal et al., 2007).

## 2 Pubertal effects on white matter development

To date, almost all developmental MRI studies of white matter development have investigated the effects of chronological age on brain structure, without accounting for the potential impact of other concurrent physiological processes that occur during adolescence (Ladouceur et al., 2012). Chronological age can be considered as a composite measure of development that incorporates a multitude of different social, physiological and psychological exposures. It has been hypothesised that the brain development observed in adolescence is significantly related to the hormonal influences that control the onset of and progression through puberty (Giedd et al., 1999, Lenroot et al., 2007, Peper et al., 2011, Sowell et al., 2002). Puberty is the process by which sexual maturity and reproductive capacity are achieved. It encompasses two distinct hormonal processes: adrenarche, the activation of the zona reticularis of the adrenal gland, and gonadarche, the activation of the gonads. These two processes trigger a rise in the production of pubertal hormones, particularly sex steroid hormones such as testosterone, dehydroepiandrosterone (DHEA) and oestradiol, resulting in physical changes such as linear and organ system growth, development of the gonads and emergence of secondary sexual characteristics, as well as changes in body proportion and facial bone structure (Verdonck et al., 1999, Lee and Houk, 2006, Meindl et al., 2012). It has been proposed that puberty may mediate changes in brain structure and function (Patton and Viner, 2007, Steinberg, 2007, Paus et al., 2008, Forbes and Dahl, 2010), and that differences in the developmental trajectories of white matter development between the sexes during adolescence, with more protracted and extensive increases in white matter volume in boys compared with girls, may reflect the different hormonal exposures and differences in pubertal timing observed between males and females (Lenroot et al., 2007, Blakemore et al., 2010).

Only two studies have investigated whether pubertal factors also influence white matter microstructure in adolescence, in addition to effects of age. The first study looked at RD in white matter tracts in males and females aged 8–28 years (*n* = 114, 63 females), and explored whether pubertal effects were present in tract regions of interest that showed significant age effects (Asato et al., 2010). Several association and projection tracts across the brain demonstrated continued immaturity (that is, a relatively high RD) in early and mid-puberty, suggesting that pubertal changes and white matter maturation may be more tightly coupled than previously thought. A second study (*n* = 77, 39 female; ages 10–16 years) reported increased FA in boys in cortico-spinal, long-range association and cortico-subcortical white matter, and reduced MD in frontal and temporal white matter compared with girls, and found that pubertal hormones such as testosterone explained variation in microstructure within some white matter regions (Herting et al., 2012). Other supporting evidence for pubertal effects on white matter in humans comes from studies looking at white matter volumetric and density changes. It has been shown that white matter density in frontal, parietal and occipital lobes increases with pubertal maturation in boys only, but decreases in cortico-spinal tracts (Perrin et al., 2009).

There is also evidence from animal studies concerning the role of puberty and pubertal hormones in brain development. These studies, mostly conducted in rodents, have provided evidence that adolescence is the second of two windows of sensitivity of the brain to sex steroids such as testosterone (Schulz and Sisk, 2006, Juraska et al., 2013). This framework, referred to as the ‘organisational-activational hypothesis’, involves an initial transient rise in testosterone during prenatal or early postnatal development which masculinises neural circuits in males, the absence of which in females results in development of a feminine phenotype. Later on, during puberty, testosterone and oestradiol are produced following gonadal maturation, and act upon sexually dimorphic neural circuits to facilitate sexually specific behaviours (Schulz et al., 2009, De Lorme et al., 2012). Of note, studies actively manipulating hormone levels are limited to animal models, which may not adequately capture the full complexity of human hormonal changes in childhood and adolescence. Puberty in humans incorporates not just gonadarche (activation of the gonads at the end of childhood) but also adrenarche (activation of the adrenal gland to produce androgens), which is not evident in rodents, and which may also relate to brain maturation (Nguyen et al., 2013).

In the current study, we sought to investigate pubertal effects on white matter microstructure in boys, and to examine to what extent these effects these can be dissociated from correlations with chronological age. Since age and pubertal developmental are tightly coupled with a high degree of shared variance, removing significant effects of age when looking at DTI changes associated with puberty may mask potentially valuable results. We sought to minimise this co-linearity and therefore maximise our ability to detect pubertal changes on white matter microstructure by using a narrow age range at a developmental stage during which a full range of pubertal stages is seen. Since the timing of puberty and the hormones associated with physiological changes differ between sexes, we focused on exploring this question in males only. We hypothesised that boys in later stages of puberty would show higher FA and lower MD than boys in earlier stages of puberty. Based on findings of age-related changes in white matter microstructure, which show widespread development in adolescence (Tamnes et al., 2010, Bava et al., 2010), we did not hypothesise region-specific changes of white matter microstructure associated with puberty, but instead were interested in ascertaining if pubertal development might explain some of the widespread developmental changes previously associated with age.

Given the correlation between pubertal development and age, we subsequently sought to disentangle these two explanatory variables by comparing regression models considering both factors and their interaction term, and we predicted that puberty would explain additional variance over and above that explained by age alone.

## 3 Methods

### 3.1 Participants

The sample consisted of 61 boys aged 12.7–16.0 years. Within this age range, typically developing males can be at any stage of puberty from Tanner 1 (pre-pubertal) to Tanner 5 (post-pubertal). In this way maximal pubertal variation was obtained while minimising age differences, so as to facilitate identification of pubertal effects. Participants were recruited via advertisements in schools, the local community, universities and hospitals.

Potential participants were excluded if they had a history of prematurity (<34 weeks gestation), a known neurological, psychiatric or endocrine disorder, or previous neurosurgery. In addition, participants were screened for any contraindications to MRI scanning. Informed consent for participation was obtained from each participant's parent or legal guardian, and participants provided informed assent for the study. Subjects received travel expenses, and were reimbursed up to a maximum of £10/h for their participation. The study was approved by the local University Ethics Committee and the National Research Ethics Committee. To ensure that puberty groups were matched on cognitive ability, participants completed the Vocabulary and Matrices subscales of the Wechsler Abbreviated Scale of Intelligence (WASI) (Wechsler, 1999), which was used as an estimation of IQ. IQ data were unavailable for two participants. Height and weight were recorded for the majority of participants and body mass index (BMI) was then calculated.

### 3.2 Puberty measures

Pubertal development was assessed through a self-report pictorial questionnaire (Taylor et al., 2001) according to established Tanner criteria (Tanner and Whitehouse, 1976). Such self-report measures have been shown to be a valid method for pubertal development assessment, with adolescents being reasonably accurate observers, especially where allocation is into two categories of early and late puberty and measures of gonadal and pubic hair stages are combined (Shirtcliff et al., 2009). In our study, participants were divided into two groups: early-mid puberty (≤Tanner Stage 3 in both pubic hair and gonadal development) (*n* = 22) and late-post puberty (≥Tanner Stage 4 in either pubic hair or gonadal development) (*n* = 39). This method has been used previously by our group (Goddings et al., 2012); from here on we shall refer to the two groups as ‘early puberty’ and ‘late puberty’.

In addition, we also collected salivary hormone assays for testosterone, dehydroepiandrosteone (DHEA) and oestradiol (each measured in pg/ml). On the morning of their scan, participants provided a saliva sample of up to 3.5 ml, collected by passive drool using a specialised collection kit developed by Salimetrics Europe Ltd (www.salimetrics.com). This unstimulated sample was collected first thing in the morning after waking, before teeth-brushing or eating and drinking (except water). For a small number of participants (*n* = 6), the saliva sample had to be collected subsequent to the scan. In these cases, the samples were collected following the same procedure within 2 weeks of the MRI scan. Samples were transported on ice, in an insulated polystyrene box, on the day of collection to the scanning centre and stored at −80 C. Samples were later analysed simultaneously by Salimetrics Europe Ltd. Saliva samples were not available (either not provided or unable to be processed due to insufficient volume) for 5 individuals for testosterone and DHEA (*n* = 56 available samples), and 8 individuals for oestradiol (*n* = 53 available samples).

### 3.3 Image acquisition

MRI data were acquired using a 1.5 T Siemens Avanto head MRI scanner with a 32-channel head coil. Head movement and scanner noise were minimised using soft cushions to support the participant's head within the head coil. Diffusion weighted images were acquired using a standard Siemens DTI echo planar imaging (EPI) sequence; gradient encoded pulses were applied in 64 directions, with AP-phase encoding, in addition to the collection of a b0 (non-diffusion weighted) image. The following parameters were used: TR = 7500 ms, TE = 104 ms, FOV = 224 mm^2^. 46 contiguous axial slices were acquired, with an isotropic voxel size of 2.3 mm^3^, and a *b*-value of 1000 s/mm^2^.

### 3.4 Image processing

DTI data were pre-processed using tools from the FDT (Functional MRI of the Brain (FMRIB) Diffusion Toolbox) part of FSL (FMRIB Software Library) (Smith et al., 2004, Woolrich et al., 2009), initially undergoing correction of eddy currents, head motion and magnetic field inhomogeneities via a 12 parameter affine registration to the first non-diffusion weighted (*b* = 0) volume. The parameters used in this correction were also applied to the diffusion gradient vectors such that both the image data and the vector data were appropriately aligned. In addition, motion-related parameters in each of the 6 possible aspects (*x, y, z*, pitch, roll, yaw) were acquired and used to calculate a summary motion parameter indicating the extent of subject head motion between each volume acquisition (Jenkinson, 1999). Subjects with a high mean relative motion of >2 mm, a previously recommended threshold (Yendiki et al., 2013), were excluded prior to statistical analysis (*n* = 1). Brain extraction (skull-stripping) was performed using FSL's Brain Extraction Tool (BET) (Smith, 2002). A diffusion tensor model was fitted to the data in a voxelwise fashion to generate whole-brain maps of the three orthogonal eigenvectors and eigenvalues, and these were used to calculate the summary measures of MD and FA. Maps of axial diffusivity (the principal eigenvalue) and radial diffusivity (the average of the remaining two eigenvalues) were stored for post hoc analyses.

### 3.5 Statistical analysis

Voxelwise statistical analysis of the DTI data was carried out using TBSS (Tract-Based Spatial Statistics) (Smith et al., 2006), part of FSL. All participants’ FA data were aligned into a common space using the nonlinear registration tool FNIRT (Andersson et al., 2010), which uses a b-spline representation of the registration warp field (Rueckert et al., 1999). This mean FA image was created and thinned to create a mean FA skeleton, which represents the centres of all tracts common to the group. Each participant's aligned FA data were then projected onto this skeleton to create a skeletonised FA image, and the same transform was applied to the MD data to create a skeletonised MD image. Skeleton voxels with a mean FA of ˂0.2 were excluded to reduce partial volume effects. The skeletonised FA and MD data for all participants were entered into voxelwise between-subject statistical analysis.

### 3.6 A priori TBSS voxelwise analysis of pubertal group

The a priori hypothesis that there would be a significant effect of pubertal group on white matter microstructure was tested on the FA and MD maps using a Student's *t*-test. Assessment of significance was by non-parametric permutation tests using the randomise tool in FSL (Nichols and Holmes, 2002; Winkler et al., 2014) with 5000 permutations and the threshold-free cluster enhancement (TFCE; Smith and Nichols, 2009) correction for multiple comparisons. Our threshold for significance after TFCE correction (*α*) at a whole brain level was 0.05.

For regions of the skeletonised maps where a main effect of puberty was identified, anatomical location was identified using the Johns Hopkins University White Matter atlas, available through FSL. The number of voxels lying within each named tract that demonstrated a significant effect of pubertal group was also calculated.

### 3.7 Post hoc regression analyses

#### 3.7.1 Puberty and age

For clusters identified in which pubertal group was significantly related to either FA or MD, the cluster-averaged FA or MD values were extracted, allowing application of three post hoc linear regression models to the data. Specifically we modelled: (1) a confirmatory analysis of the main effect of puberty without considering an effect of age; (2) an analysis of the main effect of age without considering pubertal group; and (3) an analysis of the main and interaction effects of age and puberty, taking into account their shared variance. Age was mean-centred prior to regression modelling. All regression models and group-based *t*-tests were computed using IBM SPSS Statistics for Windows, Version 20.0.

In light of recent concerns that apparent group differences in DTI indices may sometimes be driven by group differences in head motion, a motion parameter variable was also added to the regression models to confirm that head motion did not contribute significantly to group differences (Yendiki et al., 2013). The best fit regression model was identified through calculation of Akaike Information Criterion (AIC) values (Burnham and Anderson, 2002). The model with the lowest AIC is the best fitting model and is used as the reference model (with an AIC difference of 0; the numerical difference between the AIC of each model can be used to compare models. If the AIC difference was >5.9 (equating to an Akaike weight of the poorer model of <0.05), then the model with the smaller AIC was considered a significantly better fit to the data. This selection criterion has been previously used with regard to MRI structural data (Goddings et al., 2014).

#### 3.7.2 Contributions of axial and radial diffusivity to MD and FA parameters

As axial diffusivity (AD) and radial diffusivity (RD) values are used in the calculation of both FA and MD, and are therefore necessarily correlated with them, we opted not to investigate them as a priori hypotheses. Instead, we chose to investigate their influence on any regions that showed a significant relationship between either MD or FA and pubertal maturation, to help characterise the changes being measured. For any significant clusters in the FA and MD a priori TBSS analysis of pubertal group on white matter microstructure we correlated the four DTI parameters (FA, MD, AD and RD) with each other to see if there was a differential contribution of each aspect of the diffusion tensor to the result.

#### 3.7.3 Puberty and salivary hormone level

We conducted further post hoc regression analyses to investigate, in clusters where a main effect of pubertal group was identified, whether MD or FA were significantly related to pubertal salivary hormone levels. We assessed whether MD or FA were correlated with salivary levels of testosterone, DHEA and oestradiol. For hormones where this indicated a significant relationship between the hormone and the DTI parameter, we investigated whether inclusion of that salivary into the regression model improved the model fit.

#### 3.7.4 Investigation of outliers

For clusters demonstrating a significant effect of pubertal group, we assessed the extracted DTI parameter values for any evidence of outliers. MD or FA values identified following the analysis that were >3 standard deviations (SD) from the mean were considered possible outliers. Both the post hoc regression analyses and the initial TBSS analysis were repeated after excluding any such participants to assess their impact on our results.

## 4 Results

### 4.1 Participant demographics

Age, IQ and head motion in the scanner for each group are shown in Table 1, in addition to pubertal salivary hormone levels. The median Tanner stage in the early puberty group was 2, and in the late puberty group was 4. All 5 stages of pubertal development were represented. The early and late puberty groups differed significantly in age, Tanner stage, head movement and their salivary testosterone levels (all *p* values < 0.007, the Bonferroni corrected level for *α* = 0.05 and 7 tests), but did not significantly differ in terms of their IQ, BMI, DHEA or oestradiol levels (all *p* values > 0.1).

**Table 1:** Participant details with group demographics and hormone levels are shown below. Two-tailed t tests were performed to compare group differences unless otherwise stated. ^#^values for DHEA are Median and Interquartile range respectively, and a Mann Whitney *U* test was used to explore group differences in DHEA, since the distribution of this measure was positively skewed. SD = Standard deviation. Bold *p* values indicate significance at *p* < 0.05. Sample sizes are indicated for each measure where they are less than the total sample.

*F*	Early puberty (*n* = 22)	Late puberty (*n* = 39)	Test statistic	*P* value
	Mean (SD)	Mean (SD)		
Age, years	13.7 (0.72)	14.4 (0.89)	*t* = −3.29	*p* = 0.002
IQ (*n* = 59)	108 (14.2)	114 (10.6)	*t* = −1.59	*p* = 0.12
Testosterone, pg/ml (*n* = 56)	63.5 (20.8)	120 (42.4)	*t* = −6.65	*p* < 0.001
DHEA^#^, pg/ml (*n* = 56)	112 (94.4) ^#^	131 (98.8) ^#^	*U* = 317^#^	*p* = 0.5^#^
Oestradiol, pg/ml (*n* = 53)	1.51 (0.69)	1.74 (0.12)	*t* = −1.12	*p* = 0.3
BMI (*n* = 48)	21.0 (4.1)	19.9 (2.7)	*t* = 1.12	*p* = 0.3
Head movement (mean volume-to-volume displacement, mm)	0.90 (0.21)	0.75 (0.19)	*t* = 2.92	*p* = 0.005

### 4.2 *A priori* TBSS voxelwise analysis of pubertal group

#### 4.2.1 FA

Use of TBSS to conduct a whole brain analysis of early puberty versus late puberty showed that there were no significant between-group effects on FA. Although FA tended to be higher in the late puberty group compared with the early puberty group, there were no regions that survived the whole brain correction at *p* < 0.05 for either increasing or decreasing FA with advancing pubertal stage. This negative finding persisted when the threshold was lowered, and there were no significant between-group effects above a threshold of *p* = 0.2 for increased FA with increasing pubertal stage, and *p* = 0.9 for decreased FA with increasing pubertal stage. Therefore no further analyses were conducted on FA.

#### 4.2.2 MD

**Fig. 1:**
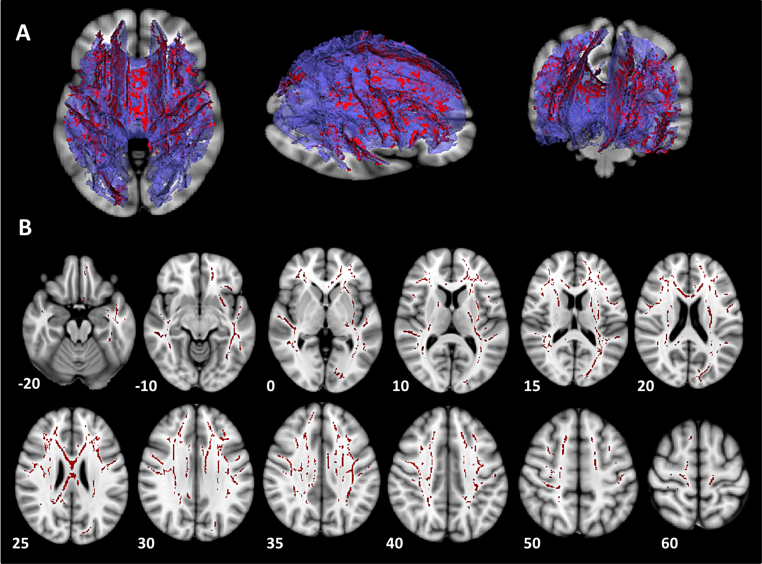
White matter regions demonstrating a significant effect of pubertal status on mean diffusivity (MD). (A) A large single cluster of voxels from the mean skeleton was identified which showed a decrease in MD in the late puberty group compared with the early puberty group. 3D images in axial, sagittal and coronal dimensions, showing areas demonstrating a significant effect of puberty on MD (red), superimposed on 3D reconstruction of the mean white matter tract skeleton (purple). These images are superimposed onto a 2D brain slice in MNI space for orientation purposes at *z* = −2, *z* = −8 and *y* = −35. Images created using Slicer (www.slicer.org) (Fedorov et al., 2012). B) Conventional display of axial slices depicting mean skeleton regions demonstrating a significant effect of puberty (red), shown in MNI space on an MNI standard brain template (MNI z coordinates are indicated for each axial slice).

When considering MD, we found evidence of a main effect of puberty that was significant at a whole brain level (*p* < 0.05 corrected). Specifically, there was a decrease in MD from early puberty to late puberty in a large single cluster comprising a number of anatomical tracts (see Fig. 1; mean cluster MD for early puberty group: 0.778 × 10^−3^ mm^2^/s, late puberty group: 0.753 × 10^−3^ mm^2^/s). White matter regions within the significant cluster demonstrating a pubertal effect are summarised in Table 2 and include those in association tracts such as the superior and inferior longitudinal fasciculus, cortico-subcortical (limbic) tracts such as the uncinate fasciculus that connects subcortical regions such as the hippocampus and amygdala with orbitofrontal cortex, and projection tracts such as the cortico-spinal tracts.

**Table 2:** Anatomical tracts included in significant cluster. A single significant cluster demonstrating a group difference between early and late puberty groups was identified using TBSS; for information purposes anatomical tracts comprising this cluster are detailed below, with the number of voxels included in the cluster detailed for each tract. MD was significantly lower in the late puberty group than the early puberty group. L; left, R; right.

Tract name		No. of voxels demonstrating significant effect of pubertal status within this tract
Superior longitudinal fasciculus (temporal part)	L	63
	R	202
Superior longitudinal fasciculus	L	3047
	R	2306
Inferior longitudinal fasciculus	L	1637
	R	905
Corticospinal tract	L	718
	R	1015
Uncinate fasciculus	L	435
Inferior fronto-occipital fasciculus	L	1458
	R	1099
Anterior thalamic radiation	L	1084
	R	669
Cingulum (cingulate gyrus)	L	567
	R	438
Forceps minor		1660
Forceps major		810
Cingulum (hippocampus)	L	18
	R	21

### 4.3 Post hoc regression analyses

#### 4.3.1 Modelling puberty and age effects on MD

**Fig. 2:**
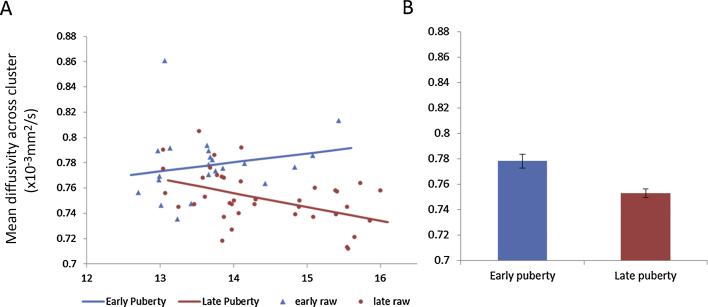
The Interaction model of how Mean Diffusivity (MD) within the single significant cluster shown in Fig. 1 changes with age and pubertal stage. The interaction model (age + puberty + age × puberty) was shown to fit the data best. Boys in early puberty (Tanner stage ≤ 3) are indicated in blue and do not show the expected decrease in mean diffusivity as age increases, in contrast boys who have progressed into late puberty (Tanner stage ≥ 4), shown in red, undergo a reduction in mean diffusivity as age increases. Lines show model fit, markers depict individual participants’ data. (B) Mean values together with bars representing standard error are shown for mean diffusivity in the significant cluster associated with puberty for the early puberty and late puberty groups.

Despite the relatively narrow age range in our sample, there remained a significant age difference between our early puberty and late puberty groups (Table 1). Therefore, we used regression models to assess whether the whole brain voxelwise pubertal effects described above could simply be due to age differences. We compared three models: a puberty only model, an age only model and an interaction model (puberty + age + age × puberty) and found that the model of best fit was the interaction model, indicating that information regarding both measures could best explain MD across the two groups (Table 3). After the interaction model, the puberty only model was the next best fit, followed by the age only model, which was the poorest fit (Table 3). In order to illustrate the best fitting interaction model, this model is plotted graphically alongside the raw and group mean MD data in Fig. 2.

**Table 3:** Comparison of regression models. The best fitting model (i.e. that with the lowest AIC) was the interaction model (Model 3) and was therefore set to be the reference model (in bold). The relative fit of other models was compared to this best model by comparing differences between the AIC for each model. The next best fitting model was that with puberty alone, and finally the lowest ranking model was that with age alone. *R*^2^ values are included for each model. Unstandardised (B) and standardised (β) coefficients, as well as the standard error of the unstandardised coefficient (SE B) are shown for each variable.

Variable	Model 1			Model 2			Model 3		
	Puberty only			Age only			Interaction		
	*B*	SE B	*β*	*B*	SE B	*β*	*B*	SE B	*β*
Puberty	−0.025	0.006	−0.48***				**−0.260**	**0.007**	−0.48***
Age				−0.110	0.004	−0.36**	**0.007**	**0.007**	**0.24**
Puberty × age							**−0.018**	**0.008**	−0.50*
*R*^2^	*0.227*			*0.132*			***0.332***		
*AIC difference*	*3.76*			*11.2*			***0 (reference)***		

* *p* < 0.05.

** *p* < 0.005.

*** *p* < 0.001.

The addition of the motion parameter to each of the three models confirmed that head motion did not improve the fit of any of the regression models, as the AIC increased (indicating a less well-fitting model) when motion was included in each of the models (AIC difference between interaction model and interaction model incorporating motion: 2.45; AIC difference between puberty model and puberty model incorporating motion 2.29; AIC difference between age model and age model incorporating motion: 0.5). This indicates that MD differences were not driven by motion artefact.

#### 4.3.2 Investigation of axial and radial diffusivity

We extracted average values of AD and RD from the single significant cluster that showed pubertal differences in MD and correlated them with the average MD value. Within this cluster both AD and RD were positivity correlated with MD (AD: *R* = 0.825, *n* = 61, *p* < 0.001; RD: *R* = 0.961, *n* = 61, *p* < 0.001) and with each other (*R* = 0.638, *n* = 61, *p* < 0.001). All three measures of diffusivity were negatively correlated with FA (MD: *R* = −0.781, *n* = 61, *p* < 0.001; AD: *R* = −0.295, *n* = 61, *p* = 0.021; RD: *R* = −0.921, *n* = 61, *p* < 0.001).

When FA, AD and RD were included in the age and puberty regression models in place of MD we replicated the same pattern of results: an interaction model always provided the largest AIC with the next best model being Model 1 (puberty alone) and finally Model 2 (age alone). For AD and RD all three models were significant with *p* values <0.005, while for FA only Models 1 and 3 (puberty alone and the interaction model) were significant (*p* < 0.05). Given how strongly the four DTI measures correlate with each other, and the fact that their inclusion did not change our interpretation of our regression model, we did not continue to analyse FA, AD and RD for the remaining models.

### 4.4 Hormonal variation and puberty group differences

**Fig. 3:**
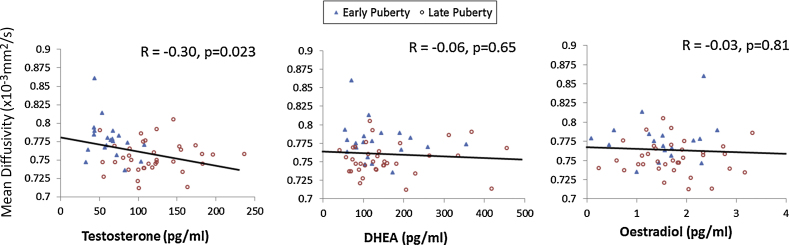
The relationship between hormonal levels, pubertal status and mean diffusivity in white matter regions that showed a significant effect of pubertal status. Scatter plots of MD within the pubertally significant regions are shown for testosterone, DHEA and Oestradiol. Markers indicate individual data, black lines indicate linear regression lines, *R* and *p* values for Pearson's correlations are shown also. Significant correlation between MD and salivary hormone level was seen only for testosterone.

To assess whether the pubertal effects on white matter tracts were related to salivary hormone levels, we investigated whether there was any correlation between MD and hormone level. We found a significant correlation between salivary testosterone and MD (*R* = −0.30, *n* = 56, *p* = 0.023) with a decrease in MD as testosterone increased. There was no relationship with MD for either DHEA or oestradiol (Fig. 3). Addition of testosterone to the puberty only and interaction regression models of MD development did not improve the fit of either model.

### 4.5 Investigation of possible outliers

Closer examination of the extracted MD values showed that one individual in early puberty had considerably higher MD than the rest of the early puberty group (>3 SD above the mean extracted MD value). As detailed in Section 3, to ensure that this participant's high MD was not driving our regression models for age and puberty, we repeated the regression after removing them from the analysis (*n* = 60). The model fit comparisons after excluding the outlier were the same as reported before, with the interaction model (standardised coefficients (*n* = 60): puberty *β* −0.51; age *β* 0.48; puberty × age *β* −0.75) representing a better fit to the data than the puberty alone model (standardised coefficients (*n* = 60): puberty *β* −0.46: AIC difference from interaction model 9.33), which in turn was still a better fit than the age alone model (standardised coefficients (*n* = 60): age *β* −0.33: AIC difference from interaction model 16.4). Again, none of the models excluding the outlier was improved by the addition of a motion term.

In addition, we also repeated the entire TBSS analysis without this participant. There was a very high degree of overlap between the regions identified which demonstrated a main effect of puberty, although there was a reduction in the whole brain corrected significance level of our findings from *α* = 0.05 to a trend level of *α* = 0.1. After re-running the TBSS analysis, we repeated the regression models once more for age and puberty on the extracted MD data, similarly finding qualitatively identical results. Again the interaction model demonstrated the best fit (standardised coefficients (*n* = 60): puberty *β* −0.65; age *β* 0.39; puberty × age *β* −0.62), the next best fitting model was the puberty only model (standardised coefficients (*n* = 60): puberty *β* −0.61: AIC difference from interaction model 8.71) and the poorest fitting model was the age only model (standardised coefficients (*n* = 60): age *β* −0.37: AIC difference from interaction model 28.9). Once again, the addition of the motion variable reduced the fit of each the models. Repeat correlation after repeating TBSS without outlier demonstrated a significant correlation between MD and salivary testosterone (*R* = −0.36, *p* = 0.007) and but no correlation between MD and either DHEA or oestradiol (both *p* > 0.3).

These analyses support our previous conclusion that our data demonstrate the existence of pubertal effects on white matter microstructure that are not simply ascribable to age, and that they are not driven by outliers.

## 5 Discussion

In the current study, we identified a main effect of pubertal status on MD in a diverse set of white matter regions, in which MD was reduced in male adolescents with more advanced pubertal development compared to their less well-developed peers. In keeping with our a priori hypotheses, the effect of pubertal status on MD was not region specific, and the significant cluster in our analysis incorporated voxels from many different anatomical tracts. These pubertal effects were distinct from age-related differences in the same regions, and the best fitting model to explain the differences in MD incorporated both age and puberty measures. In addition, there was a partial relationship between MD in the regions significantly associated with pubertal status and salivary testosterone, such that higher testosterone levels were associated with decreased MD.

### 5.1 Comparison with previous DTI findings in adolescence

In the current study we did not find pubertal effects on FA. Although increases in FA during adolescence have been found in studies looking at white matter changes with increasing age, these studies have typically been conducted over a much wider age range than our study, often showing increases in FA from late childhood into the third decade of life (Ladouceur et al., 2012). Over the narrow age range within our study (3.3 years), pubertal development was associated with a significant reduction in MD but no change in FA.

Although the DTI studies in adolescence described above have shown that in general increases in FA tend to occur in association with decreases in MD, these changes are not necessarily reciprocal, that is inversely equivalent, in nature (Tamnes et al., 2010, Lebel and Beaulieu, 2011). Detailed comparisons of changes in FA and MD during the course of adolescence have shown that MD tends to change predominantly in frontal, temporal and medial parietal lobes, whereas FA changes most strongly in lateral parietal, lateral occipital and frontal regions (Tamnes et al., 2010). These anatomical differences between FA and MD developmental trajectories suggest that each parameter differently captures development of white matter microstructure. In addition, FA and MD were differentially related to cortical thickness and white matter volume measures, again indicating differences between the microstructural changes best characterised by each measure.

### 5.2 Previous DTI studies of puberty and white matter changes

The present study focused specifically on the pubertal effects on white matter maturation. Two previous DTI studies have reported pubertal-associated changes, but these have been in the context of wider investigations of age effect on white matter microstructure. While these previous studies employed different analytical approaches, both initially accounted for effects of chronological age, thereby removing any shared variance between age and puberty from the remaining analysis concerning pubertal effects on brain structure. The first studied a cross-sectional sample of young people between 8 and 28 years (males and females), reporting radial diffusivity (RD), a measure of diffusion in the direction perpendicular to the predominant direction of diffusion (Asato et al., 2010). This study identified a diverse set of 19 regions in which there was a significant effect of age on RD, finding a reduction in RD with increasing age, then explored whether this age effect could be accounted for by pubertal maturation.

Similar to our findings, Asato et al., found continued decreases in the diffusion measure into post-puberty, though MD reflects overall magnitude of diffusion independent of direction, whereas RD considers diffusion only in the direction perpendicular to the predominant diffusion direction. This reduction in RD occurred across several major tracts including the superior longitudinal fasciculus, the uncinate fasciculus and the anterior thalamic radiation. Asato et al., also found evidence for age-by-sex-by-pubertal stage interactions, suggesting that RD in these regions varied with both age and pubertal stage in different ways for boys versus girls. In addition, all but one of the 19 clusters showed an immature pattern in both the early and mid-pubertal stages, i.e. RD remained high, indicating that these regions within both association and projection tracts only became ‘adult-like’, with a lower RD, within the post-pubertal stage.

The second DTI study investigating puberty included a sample of males and females aged 10–16 years (Herting et al., 2012). Herting and colleagues found a significant relationship between FA and puberty in only a single white matter region (the right insular gyrus), after co-varying for age (and thereby removing any shared variance between age and puberty, which are tightly coupled). They found no relationship between MD and pubertal score after co-varying for age. When investigating the relationship between sex steroids and DTI measures, again after co-varying for age, the authors reported a significant relationship between testosterone and FA (in regions including the superior temporal gyrus, corpus callosum and superior frontal gyrus) and MD (in the superior frontal gyrus).

In line with the findings of Herting et al., we also found evidence for a relationship between testosterone and white matter microstructure changes during adolescence, although we found that pubertal development was related to changes in MD rather than FA. These discrepancies could relate to a number of methodological differences between the studies, including different age ranges (3 years in our study compared with 6 years in Herting et al.), method of assignment of pubertal group/score (self-report pictorial questionnaire versus the Peterson Developmental Scale (Petersen et al., 1988)), and hormone sampling method (blood versus salivary assays).

### 5.3 Evidence for pubertal influences on white matter microstructure

There is evidence for pubertal effects on brain development from both human and animal studies. A review summarising the effects of pubertal hormones on connectivity in the human brain concluded that sex steroids such as testosterone and oestradiol play a key role in both the initial organisation of structural connections in utero, and in activating areas linked by such connections later on in life during adolescence (Peper et al., 2011). For example, there is evidence from electroencephalographic (EEG) data and functional connectivity MRI data for a role for testosterone in decreasing subcortical-cortical connectivity, and increasing connectivity between sub-cortical areas (Schutter and van Honk, 2004, Miskovic and Schmidt, 2009, van Wingen et al., 2010, Volman et al., 2011). Whilst it is difficult to extrapolate these functional studies (particularly for EEG given its high temporal but low spatial resolution) to our DTI results demonstrating white matter changes in specific regions, these data at least indicate that sex steroids such as testosterone can impact on brain function.

In our sample of adolescent boys, we found correlations between DTI measures and salivary testosterone, but neither of the other sex hormones, during adolescence. This may reflect a greater importance of testosterone than other hormones in white matter development during puberty in males. Testosterone is the primary androgen released from the gonads in males, and shows the greatest rise across puberty, being 45 times higher in adult men than in pre-pubertal boys (Biro et al., 1995). Sex steroids such as testosterone play a critical role specifically in white matter development during adolescence, acting as trophic factors impacting on development of axons themselves and also their supporting cells (McEwen et al., 1982, Melcangi et al., 2001, Peper et al., 2011). It is known from both animal and post-mortem human studies that myelination continues during adolescence and is more extensive in males than females (Yakovlev and Lecours, 1967, Kim and Juraska, 1997), possibly underlying the larger increases in white matter volume seen in human males than females during adolescence (Lenroot et al., 2007).

There is also evidence that sex steroids are important in determining diverse structural characteristics within the brain including white matter microstructure, for example affecting synapse number, neurite outgrowth, dendritic branching and myelination (Jordan and Williams, 2001, Melcangi et al., 2001, Romeo, 2003, Cooke and Woolley, 2005, Garcia-Segura and Melcangi, 2006). Mechanisms by which sex hormones exert such control include their action as transcription factors for gene expression and cellular proliferation (Melcangi et al., 2001), and activation of signalling cascades (Nguyen et al., 2005). Sex steroids can also directly impact glial cells and thereby influence myelination (Garcia-Segura and Melcangi, 2006), and a role for testosterone and oestradiol as neuro-protective agents in demyelination disorders such as multiple sclerosis and in spinal cord nerve injury is being advocated (Leonelli et al., 2006, Melcangi and Mensah-Nyagan, 2006, Gold and Voskuhl, 2009). Furthermore, gonadectomy led to demyelination of the corpus callosum in intact, gonadectomised and hormone-replaced gonadectomised rats, and this effect was more deleterious in males than females (Patel et al., 2013).

The importance of the androgen receptor with respect to modulation of brain structure and function is supported by animal work confirming its presence within axons, dendrites and glial cells within cortical regions, in a regionally-specific manner (Sarkey et al., 2008). In addition, studies in human adolescents have found an association between testosterone level and increasing white matter that is modulated by a functional polymorphism in the androgen receptor gene, the short arm polymorphism of which leads to more efficient androgen signalling than the long arm polymorphism. Increased testosterone levels had a more robust effect on white matter volume in boys with the short arm androgen receptor polymorphism (Paus et al., 2010, Raznahan et al., 2010), and this was associated with an increased likelihood of depression in adolescence (Perrin et al., 2008).

In summary, there is evidence from both animal and human studies for pubertal effects on brain structure, including white matter microstructure, and for mechanisms by which pubertal hormones such as testosterone might mediate such changes. Our finding that pubertal development is associated with changes in white matter characteristics is consistent with this. These changes were partly correlated with testosterone, although addition of testosterone to the regression models did not improve their fit. Pubertal group and testosterone were highly correlated in our sample, and were therefore capturing similar aspects of pubertal maturation. Addition of testosterone to the regression models was therefore likely to be redundant, and reduced the efficiency of the model.

### 5.4 Interpretation of DTI parameter results as indices of white matter microstructure

In our post hoc analysis of the four DTI parameters we replicated the results of a recent study of 17 adults by De Santis et al. (2014) in which MD and FA in 42 white matter regions of interest were not correlated with each other. In addition, in their study and in ours, MD and FA were both positively correlated with AD and showed opposite directions of correlation with RD (positive for MD and negative for FA).

De Santis and others found that FA but not MD showed a significant correlation with the regions’ myelin water fraction (MacKay et al., 1994, Deoni et al., 2008, De Santis et al., 2014). Rather than representing myelination, MD decreases have instead been associated with proliferation and/or growth of astrocytes in grey matter (Blumenfeld-Katzir et al., 2011, Sagi et al., 2012). We found that puberty is associated with MD more significantly than with FA. This suggests that changes in white matter microstructure associated with pubertal development may relate more to reduction in the overall magnitude of diffusion within the white matter than to changes in the directionality of diffusion. This decrease in MD is likely to indicate more cells and cell components in the white matter regions shown in Fig. 1. However, given that our findings are not specific to MD over the other DTI parameters it is possible that the decrease in MD is caused by an increase in myelin. But since MD explains more variance in age and puberty than the three other DTI parameters (FA, AD and RD) it is more likely that alternative or additional cellular processes are driving the difference we observe between early and late puberty. The exact nature of these changes cannot be elucidated with human in vivo studies.

### 5.5 Methodological considerations and future directions

While our findings are of interest in understanding differential contributions of chronological age and puberty on adolescent brain development, there are limitations to this study. Our sample is cross-sectional in nature, and longitudinal studies that assess the relationship between pubertal changes and brain development within the same individuals are needed. Despite our efforts to constrain variation in age to a minimum, we still needed to grapple with the biologically inherent co-linearity of age and pubertal development. Studies recruiting young people with constitutional delay of puberty, or conversely precocious puberty, would allow the comparison of individuals who were exactly age-matched but at contrasting stages of puberty. However, since aberrations in pubertal timing may themselves be related to additional clinical pathology such as the presence of chronic disease or an endocrine disorder, this approach may prove problematic in understanding the normal process of pubertal development. We opted to focus on males in this study: males and females experience differences in the timing of pubertal processes during adolescence, and therefore direct comparisons between the sexes are confounded by such variations in the timing of puberty and the associated hormonal influences. Future studies should additionally consider pubertal relationships with white matter development in females. Measurement of puberty presents a number of methodological challenges for research. In this study we evaluated physical pubertal development using a self-report Tanner stage questionnaire. While this is the most widely used and clinically validated measure of pubertal development, it is nevertheless a relatively imprecise measure that subjectively categorises puberty into broad developmental stages, and is subject to potential biases. Previous studies of adolescents have generally shown variable levels of correlation between physician-assessed and self-assessed Tanner stage from relatively low to very high (Coleman and Coleman, 2002).

We also need to consider the advantages and disadvantages of single salivary hormone assays. This method of hormone sampling is a validated approach that is non-invasive and thereby more acceptable to young people than a blood test (Dorn et al., 2006, Shirtcliff et al., 2009, Goddings et al., 2012). However, it is as yet unclear how peripheral hormone levels in saliva or blood relate directly to hormone concentrations that cross the blood-brain barrier, and how in situ enzymes, e.g. aromatase, affect androgen receptor exposure to circulating androgens in vivo. Using a single sample salivary assay restricts our ability to consider the variation in hormonal level both diurnally and over longer time periods within our participants across pubertal development. Further exploration of hormonal variation is being conducted by other groups at present (Mundy et al., 2013).

Finally, we note that effects of head movement within the MRI scanner have been highlighted recently as a potential confound in studies comparing DTI data between groups (Yendiki et al., 2013). In our study, head movement was increased in the early puberty compared with late puberty, suggesting that movement may be an important consideration. Unlike many previous DTI studies of adolescence and puberty, we incorporated motion parameters into our analysis, confirming that head motion had a minimal impact on our results and did not contribute to models predicting differences between the early and late puberty groups.

## 6 Conclusions

This study provides evidence to show that pubertal effects on white matter microstructure are evident in boys. These findings were not simply due to age effects, but were more closely related to an interaction effect of puberty and age, and to pubertal status alone, than to age. In addition, white matter changes were at least partially related to salivary testosterone levels in boys, indicating that these differences in white matter may be underpinned partially by hormonal processes. This study provides evidence that pubertal processes impact on adolescent white matter development in addition to effects of chronological age.

## Conflict of interest

All authors report no actual or potential conflicts of interest including any financial, personal or other relationship with other people or organisations that could inappropriately influence, or be perceived to influence this work.
